# Molecular docking analysis of mTOR protein kinase with chromatographically characterized compounds from *Clerodendrum inerme L.* leaves extract

**DOI:** 10.6026/97320630018381

**Published:** 2022-04-30

**Authors:** Dhanalakshmi Arumugam, Sasireka Ganesan, Karthikeyan Kalimuthu Ayyakkalai Marikkannu

**Affiliations:** 1 Department of Zoology, N. M. S. S. Vellaichamy Nadar College, Madurai 625019, Tamil Nadu, India; 2 Department of Zoology, Sri Meenakshi Government Arts College for Women, Madurai 625002, Tamil Nadu, India

**Keywords:** Molecular docking, chromatographic analysis, mTOR protein kinase, Clerodendrum inerme L

## Abstract

The mTOR protein is known to be linked with cancer. Therefore, it is of interest to document the molecular docking analysis of mTOR protein kinase with chromatographically characterized compounds from Clerodendrum inerme L. leaves extract. The GC-MS analysis suggested that, totally 25 bioactive compounds were present in the extract of Clerodendrum inerme. Molecular docking analysis show that the bioactive compounds such as Triethoxysilanol, Piperazine dihydrochloridehydrate, 2,4(1H,3H)-Pyrimidinedione, 5-methyl and 4',7-Dihydroxyflavanone showed good glide score and glide energy within the acceptable and permissible limits of ADME properties for further consideration in drug discovery.

## Background:

The mammalian target of rapamycin (mTOR), a member of the phosphatidylinositol 3-kinase (PI3K)-related kinase family, functions as a cardinal regulator of cell growth, proliferation, metabolism, and survival via mTORC1 and mTORC2 [1]. The biological function of mTORC1 is mainly regulated by inducing the phosphorylation of both eukaryotic translation initiation factor 4E-binding protein 1 (4EBP1) and ribosomal S6 kinase 1 (S6K1) [2], while mTORC2 mainly phosphorylates serine/threonine protein kinase AKT at the serine residue S473 [3]. mTOR is a key node in PI3K/Akt/mTOR pathway and unusual mTOR signaling can promote the development and progression of various cancers [4,5]. Thus, developing clinical drugs based on mTOR kinase inhibition is of great interest. Rapamycin and its analogs are well-known mTOR allosteric site inhibitors that bind to the intracellular immunophilin FK506-binding protein 12 (FKBP12). The resulting complex binds to the FKB domain of mTOR and differentially suppresses mTORC1-mediated phosphorylation of the S6K1 and 4EBP1 substrates [6]. Currently, the rapamycin derivatives (termed rapalogs) temsirolimus (CCI- 779) and everolimus (RAD-001) are approved by the FDA for the treatment of renal cell carcinoma [7]. However, rapalogs exhibit therapeutic effects in a relatively limited number of tumor models, which is partly attributed to the fact that rapalogs block only the C1 isoform and are resistant to mTORC2. Moreover, rapalogs can activate PI3K signaling through a negative feedback loop [8], which might significantly reduce mTORC1 anti-cancer activity [9]. Evidence suggests that mTORC2 also participates in tumor growth and survival [10]. To find a broad and robust anti-cancer treatment, ATP-competitive mTOR kinase inhibitors targeting both mTORC1 and mTORC2 have to be developed. In addition, ATP-competitive mTOR inhibitors can prevent the activation of the negative feedback loop [11], resulting in therapeutic advantages over rapalogs [12]. Some highly potent and selective ATP-competitive mTOR inhibitors has been discovered and are rapidly moving into clinical trials, e.g., AZD8055, AZD2014, OSI-027, and INK- 128 [13]. Although these inhibitors have been widely used as a biological tool to interrogate the mTOR signaling pathway, none of the ATP-competitive mTOR inhibitors has entered into phase III clinical trials. Hence, the need to discover novel mTOR kinase inhibitors that can be developed into therapeutic candidates for cancer treatment continues to grow [14].

Natural products are structurally diverse and have more potential druggable pharmacophores than synthetic compounds [15]. Moreover, they are the source of most of the active ingredients in medicines. Clerodendrum inerme L. (C. inerme) is a medicinal plant which belongs to the family of Verbenaceae. Its native to South and South-east Asia, Australia, and Pacific islands and widely spread in tropical and subtropical region of the world [16]. The crude extract of this plant is used in the treatment of scrofulous and venereal infections, and also as an antidote for poisoning from fish, crabs, and toadstools [17]. Phytochemical studies of this plant lead to the isolation of sterols, flavones, clerodane diterpenes, neolignans, iridoid glycosides and triterpenes. The fresh leaf juice is used externally for skin diseases. Furthermore, the roots are boiled in oil and used in rheumatic affections. It also possesses antimicrobial, anti-coagulants, uterine stimulant, hypertensive, and laxative activities [18]. Therefore, it is of interest to document the molecular docking analysis of mTOR protein kinase with chromatographically characterized compounds from Clerodendrum inerme L. leaves extract.

## Materials and Methods:

### Collection of plant material:

The plant material C. inerme was collected in the area of Coimbatore district of Tamil Nadu, India, and authenticated by Dr. P. Satyanarayana, Botanical Survey of India, Coimbatore, Tamil Nadu, India. The Voucher number is BSI/SRC/5/23/2011-12/Tech. 1538 [19]. Collected fresh plant material was cleaned, air dried and powdered.

### Extract preparation:

100 g of the whole plant powder was extracted with 500 ml of ethanol for 72 hours using exhaustive extraction procedure. The collected and concentrated extract was stored at 4°C for further studies.

### GC-MS analysis:

GC-MS analysis of ethanolic extract of C. inerme was done by Agilent technologies 7890 A. Briefly, the Helium was used as carrier gas at low down of 1.0 ml/min. The injector was functioned at 250°C and oven heat was maintained as follows: 60°C for 15 min, then slowly amplified to 280°C at 3 min. MS were taken at 70 eV; a scan distance of 0.5 seconds and fragments starts from 50 to 650 Da. Total GC operation period was 25 min. The comparative percentage amount of every module was calculated by evaluating its average peak area to the total areas, software adopted to handle mass spectra and chromatograms was a Turbo mass. The percentage composition of plant extract was calculated. Interpretation of GC-MS was done by the NIST database and Willey libraries in addition to comparison of their retention indices.

### Protein structure prediction:

Three dimensional structure of mTOR protein was retrieved through Protein Data Bank and its PDB ID: 4JT5. The protein preparation wizard (standard methods) was used to prepare the protein structure, which is accessible in grid-based ligand docking with energetics (Protein Preparation Wizard, Schrödinger, 2016). Protein was optimized and minimized by using RMSD 0.30 Å and OPLS (2005) force field.

### Active site prediction:

mTOR binding site was recognized by SiteMap module (SiteMap 5.5, Schrodinger, 2016). SiteMap computation starts with an early search step that characterizes through the grid points one or more regions on the protein surface that could be appropriate for binding ligands to the protein.

### Ligand preparation:

Identified natural compounds from the GC-MS analysis were prepared using the LigPrep 2.4 (LigPrep 2.4, Schrodinger, 2016) for the molecular docking simulations. The OPLS 2005 force field was used to optimize the ligands structure.

### ADME properties prediction:

ADME properties of mTOR inhibitors were analyzed by QikProp 2.3 module. QikProp helps to analyze the pharmacokinetics and pharmaco-dynamics properties of the ligands.

### Docking simulations:

The molecular docking simulations were carried out using Glide module (Glide 5.6, Schrodinger, 2016) by standard precision (SP) method. All the ligands were docked in to binding pocket of mTOR using. The OPLS_2005 force field was used for docking protocol. The lowest energy docked complexes were found in the majority of similar docking conformations.

## Results and Discussion:

Medicinal plants are rich source of bioactive compounds and they posses many biological activities against human diseases and disorders [20]. Characterization of phytochemicals by chromatography and spectroscopic methods could deliver the efficient information of herbal medicines [21]. GC-MS is a combined analytical method to recognize the numerous phytochemicals present in the plant extract [22]. The GC-MS analysis characterized 25 natural compounds from C. inerme extract (Table 1 - see PDF) namely; Styrene (2.32%), Triethoxysilanol (2.31%), Piperazine dihydrochloridehydrate (1.26%), 1-Tridecene (CAS) (1.26%), 2,4(1H,3H)-Pyrimidinedione, 5-methyl- (CAS) (1.11%), Cyclohexane, hexyl- (1.24%), acrylic acid octyl ester (2.98%), Benzofuran, 2,3-dihydro- (CAS) (0.87%), 7-Hexadecene, (Z)- (CAS) (3.12%), 2-Furancarboxaldehyde, 5-(hydroxymethyl)- (CAS) (4.13%), (2S)-N-(Hex-5-en-2-yl)carbamic acid tert-butyl ester (2.16%), 1-Heptadecene (CAS) (4.79%), 1-Pentanol, 2-methyl- (CAS) (4.10%), (cis)-2-nonadecene (6.82%), 3-O-Pivaloyl-5,6-O-thiocarbonyl-1,2-O-isopropylidene-à,D-glucofuranose (0.85%), (2.21%), 1-(3-Nitrophenyl) 2-nitropropan-1-ol (2.21%), (R)-10-Methyltridecene (23.81%), 4-((1E)-3-Hydroxy-1-propenyl)-2-methoxyphenol (16.59%), 4', 7-Dihydroxyflavanone (6.43%), 1-Nonadecene (1.81%), 7,10-Hexadecadienoic acid, methyl ester (CAS) (4.03%), 11,14,17-Eicosatrienoic acid, methyl ester (CAS) (1.83%), 10-Methyl-1-hexadecanol (1.02%), 2-[(p-Methoxyphenyl)hydroxymethyl]-tropone (2.11%), 9,10-Anthracenedione, 2-methyl- (0.84%). Molecular docking simulation is the significant method to analyze the binding mechanism of protein ligand complexes [23]. The docking results of selected natural compounds were complexes with mTOR protein showed in Table 2(see PDF). Among that, Triethoxysilanol, Piperazine dihydrochloridehydrate, 2,4(1H,3H)-Pyrimidinedione, 5-methyl and 4',7-Dihydroxyflavanone showed better Glide score of -7.045, -7.984, -8.087, -6.908 respectively and the glide energy was -40.237, -38.876, -42.936 and -39.874 kcal/mol respectively (Figure 2 to 6 - see PDF). ADME properties of screened compounds do not predict any adverse effect that could be implicated in the failure of drugs. Consequently, there is increasing awareness in the early prediction of ADME properties reaching success rate of compounds development with the objectives. The results, Triethoxysilanol, Piperazine dihydrochloridehydrate, 2,4(1H,3H)-Pyrimidinedione, 5-methyl and 4',7-Dihydroxyflavanone (shown in table 3) were under acceptable range.

## Conclusion:

Molecular docking analysis show that the bioactive compounds such as Triethoxysilanol, Piperazine dihydrochloridehydrate, 2,4(1H,3H)-Pyrimidinedione, 5-methyl and 4',7-Dihydroxyflavanone showed good glide score and glide energy within the acceptable and permissible limits of ADME properties for further consideration in drug discovery.

## References

[1] Laplante M, Sabatini DM (2012). Cell..

[2] Showkat M (2014). Mol Biol Int..

[3] Vadlakonda L (2013). Front Oncol..

[4] Gao Y (2016). Stem Cell Investig..

[5] Cidado J, Park BH (2012). J Mammary Gland Biol Neoplasia..

[6] Chen L (2014). Mol Divers..

[7] Yin Zheng Y, Jiang Y (2015). Mol Cell Pharmacol..

[8] Chandarlapaty S (2012). Cancer Discov..

[9] Seraina Faes S (2017). Oxid Med Cell Longev..

[10] Kim LC (2017). Oncogene..

[11] Markman B (2010). Oncotarget..

[12] Badura S (2013). PLoS One..

[13] Malley CO (2015). BBA Clin..

[14] Fouqué A (2016). Recent Pat Anticancer Drug Discov..

[15] Perumal PC (2012). Asian Pac J Trop Dis..

[16] Wang P (2012). Fitoterapia.

[17] Kanchanapoom T (2001). Phytochemistry.

[18] Ibrahim SRM (2014). Bull Fac Pharm Cairo Univ..

[19] Poornima K (2014). Biomed Res Int..

[20] Perumal PC (2014). J App Pharm Sci..

[21] Perumal PC (2015). Int J Curr Pharm Rev Res..

[22] Sowmya S (2016). Anal Chem Let..

[23] Perumal PC (2015). Pharmacog Res..

